# Pharmacy premises licensing policy formulation: experience from Ghana

**DOI:** 10.1186/s12961-021-00680-7

**Published:** 2021-02-08

**Authors:** Augustina Koduah, Reginald Sekyi-Brown, Joseph Kodjo Nsiah Nyoagbe, Daniel Amaning Danquah, Irene Kretchy

**Affiliations:** 1 Department of Pharmacy Practice and Clinical Pharmacy, School of Pharmacy, University of Ghana, P.O. Box LG43, Legon, Ghana; 2 No 001 Community 14 Annex Tema, Teshie Nungua Estates, P.O. Box TNE 644, Accra, Ghana; 3 Deputy Registrar Operations Pharmacy Council, P.O. Box AN 10344, Accra North, Ghana

**Keywords:** Ghana, Legislation, Pharmacy business, Pharmacy licence, Pharmacy premises regulation, Policy formulation

## Abstract

**Background:**

Licences to operate pharmacy premises are issued by statutory regulatory bodies. The Health Institutions and Facilities Act (Act 829) and Health Professions Regulatory Bodies Act (Act 857) regulate pharmacy premises and the business of supplying restricted medicines by retail, respectively, and this could create a potential regulatory overlap for pharmacy practice in Ghana. We theorise that the potential overlap of regulation duties stems from how law-makers framed issues and narratives during the formulation of these Acts.

**Objective:**

To describe the policy actors involved, framing of narratives and decision-making processes relating to pharmacy premises licensing policy formulation.

**Methods:**

A qualitative study was conducted and data gathered through interviewing eight key informants and reviewing Hansards, reports, bills, memoranda and Acts 829 and 857. Data were analysed to map decision-making venues, processes, actors and narratives.

**Results:**

The Ministry of Health drafted the bills in July 2010 with the consensus of internal stakeholders. These were interrogated by the Parliament Select Committee on Health (with legislative power) during separate periods, and decisions made in Parliament to alter propositions of pharmacy premises regulations. Parliamentarians framed pharmacies as health facilities and reassigned their regulation from the Pharmacy Council to a new agency. The Pharmacy Council and the Pharmaceutical Society of Ghana could not participate in the decision-making processes in Parliament to oppose these alterations. The laws’ contents rested with parliamentarians as they made decisions in venues restricted to others. Legislative procedure limited participation, although non-legislative actors had some level of influence on the initial content.

**Conclusion:**

Implementation of these laws would have implications for policy and practice and therefore understanding how the laws were framed and formulated is important for further reforms. We recommend additional research to investigate the impact of the implementation of these Acts on pharmacy practice and business in Ghana and the findings can serve as bargaining information for reforms.

## Background

Pharmacy premises are facilities in which pharmaceutical services are offered and a licence is needed to operate one [1]. The licence can be provided by a national statutory body as in many countries or at the local level in federal states [2]. In Ghana the licence is provided by government through a national statutory regulatory body. The issuance of a licence to operate pharmacy premises started with the enactment of Druggists Ordinance in 1892 by the colonial government where the governor issued licence to persons to carry on the business of mixing, compounding, preparing, selling, retailing or dispensing any drug or poison [3]. The statutory regulatory body—Board of Examiners—was then established under the Druggists Ordinance to provide the licence for pharmacy premises and practice. Since then, the name of the statutory regulatory body had evolved as laws establishing it were revised by law-makers to take into account the changing role of pharmacy profession and increasing profession influence [4]. The Board of Examiners changed to Pharmacy and Poisons Board under the Pharmacy and Poisons Ordinance 1946 [5], then to Pharmacy Board and Pharmacy Council under the Pharmacy and Drugs Act 1961 [6] and the Pharmacy Act 1994 [7], respectively. The revised laws did not create a new national statutory regulatory body but expanded the administrative capabilities and responsibilities of the existing one to regulate pharmacy premises and practice [4].

In 2011, government enacted a Health Institutions and Facilities Act (Act 829) to establish a Health Facilities Regulatory Agency (HeFRA) to license and monitor health facilities including pharmacy premises for the provision of public and private health care services. Under Act 829, the Health Facilities Regulatory Agency took over the issuance of a pharmacy premises operation licence from the Pharmacy Council. By law the regulation of pharmacy premises was ceded to a new national statutory agency. A provision was therefore made in Act 829 for the Pharmacy Council to transfer any information, knowledge, materials and staff necessary for the functioning of the agency within 5 years after the commencement of Act 829 [8].

However, in 2013 the Pharmacy Act 1994 (Act 489), the law regulating pharmacy premises and practice, was revised and incorporated as part four in a consolidated Health Professions Regulatory Bodies Act (Act 857) [9]. The revised Act excluded all statements that sought to make provisions for pharmacy premises regulation. Part four of Act 857 established a Pharmacy Council (an existing national statutory regulatory body) with added objectives among others to ensure the equitable and accessible distribution of pharmaceutical premises. Additionally, under Section 93 of Act 857, the Pharmacy Council was to grant licence to a body corporate or a government institution to carry on the business of mixing, compounding, preparing or supplying restricted medicines by retail under the supervision of a superintendent pharmacist. Act 857 defined restricted medicines as prescription only medicines, pharmacy only medicines, over the counter medicines and any other classifications approved by the Minister for Health [9]. Act 829 and Act 857 are different statutory policies regulating pharmacy premises and the business of mixing, compounding, preparing or supplying restricted medicines by retail under the Health Facilities Regulatory Agency and Pharmacy Council, respectively.

Pharmacy operators are in the business of supplying restricted medicine by retail and operate within pharmacy premises and there is therefore a seemingly overlap of regulation duties between the national statutory bodies—Pharmacy Council and HeFRA. We theorise that the potential overlap of regulation duties stems from how law-makers used their legislative power to frame issues and narratives relating to pharmacy premises and health care service provision and how they were able to influence others during the decision-making processes.

Laws regulating pharmacy premises like any other laws are enacted at national level by policy actors with powers to formulate such laws. Power approaches to policy-making process which focus on power and its distribution among actors (groups and elites) and how they shape decisions are important for understanding how policy actors use their power sources to define problems, frame issues and make decisions [10]. Power approaches view decision-making as something which is shaped and determined by the structures of power such as bureaucratic and political arrangements, pressure groups and technical knowledge [10].

The decision-making process involves steps in which choices are made or the preferred option is selected at a point or series of points in time when policy-makers define problems and propose solutions [10]. Problem definition during decision-making involves how problems are framed and how policy actors debate and interpret the issue for decision [10]. Since there is no one fixed problem definition for a particular policy issue, such policy issues are subject to the interpretative manoeuvres of powerful policy actors and their ability to propose convincing solutions [11]. Problem definitions are therefore shaped out of debates, rebuttals and meanings of narratives [12, 13]. It is therefore important to understand who is defining the problem, pushing narratives and the context in which decisions are made.

Understanding how policy actors framed issues, advanced specific narratives and made decisions relating to regulation of pharmacy premises and the business of mixing, compounding, preparing or supplying restricted medicines by retail is essential to inform pharmacy policy-making and learning. Additionally, there is little information available to practitioners who wish to understand how issues relating to pharmacy premises and business regulation were framed and debated in a low- and middle-income country (LMIC) setting such as Ghana. But this area of policy analysis is less studied. Other studies have focused on the pharmacy profession and education [14–16] and legal and regulatory framework for community pharmacy [2].

This paper aims to advance our understanding of policy formulation in a LMIC setting by exploring the decision-making processes, problem definitions and the framing of narratives leading to the formulation of statutory policies for pharmacy premises and the business of mixing, compounding, preparing or supplying restricted medicines by retail in Ghana.

## Methods

We conducted a qualitative study to examine the policy actors involved and how they framed issues during decision-making processes in the design of Act 829 and Act 857.

### Data collection methods

Data collection methods included extensive document review and key informant interviews. These data collection methods occurred concurrently to allow for an iterative process so as to validate information from the interviews, request for relevant documents from key informants and better understand the context within which these reviewed documents were written. Document review and analysis were used to examine the sequence of decision-making, trace and map events, identify the policy actors involved in the decision-making processes and how they framed issues relating to regulation of pharmacy premises and the business of mixing, compounding, preparing or supplying restricted medicines by retail. The documents reviewed and analysed are summarized in Table 1. All documents reviewed and analysed were written documents and publically available. The Acts and bills and accompanying memoranda were obtained from the Ghana Publishing Corporation. The parliamentary debates reports (Hansards) from 28 October 2010 when the Health Institutions and Facilities Bill was first read to 21 December 2012 when the Health Professions Regulatory Bodies Bill was passed in Parliament were retrieved from the Ghana Parliament Library. The Council of Elders of the Pharmaceutical Society of Ghana (PSGH) memorandum, review of the pharmacy council component of the Health Professions Regulatory Bill documents were obtained from the PSGH office. To ensure rigour, the written documents were assessed on the basis of four criteria development by Scott [17]: (1) authenticity of the documents was assessed to ensure they were genuine and of unquestionable origin; (2) credibility of the documents was assessed to ensure the documents were free from error or distortion; (3) representativeness of the documents was assessed to ensure they were typical of their kind; (4) the documents were assessed to ensure the texts were clear and comprehensible [17].

**Table 1:** List of documents reviewed

Documents	Date
Hansards	
Parliamentary Debates (Official Report) Fourth Series Vol. 71 No. 7 First reading of Bills—Health Institutions and Facilities Bill, 2010	28 October 2010
Parliamentary Debates (Official Report) Fourth Series Vol. 72 No. 39 Second reading of Bills—Health Institutions and Facilities Bill, 2010	22 March 2011
Parliamentary Debates (Official Report) Fourth Series Vol. 73 No. 22 Consideration stage of Bills—Health Institutions and Facilities Bill, 2010	23 June 2011
Parliamentary Debates (Official Report) Fourth Series Vol. 73 No. 37 Third reading of Bills—Health Institutions and Facilities Bill, 2010	20 July 2011
Parliamentary Debates (Official Report) Fourth Series Vol. 79 No. 3 Second reading of Bills—Health Professions Regulatory Bodies Bill, 2010	24 October 2012
Parliamentary Debates (Official Report) Fourth Series Vol. 79 No. 4 Consideration stage of Bills—Health Professions Regulatory Bodies Bill, 2011	25 October 2012
Parliamentary Debates (Official Report) Fourth Series Vol. 79 No. 9 Consideration stage of Bills—Health Professions Regulatory Bodies Bill, 2011	18 December 2012
Parliamentary Debates (Official Report) Fourth Series Vol. 79 No. 11 Consideration stage of Bills—Health Professions Regulatory Bodies Bill, 2011	20 December 2012
Parliamentary Debates (Official Report) Fourth Series Vol. 79 No. 12 Consideration stage of Bills—Health Professions Regulatory Bodies Bill, 2011 Second consideration stage of Bills—Health Professions Regulatory Bodies Bill, 2011 Third reading of Bills—Health Professions Regulatory Bodies Bill, 2011	21 December 2012
Memoranda accompanying the Bills	
Health Institutions and Facilities Bill, 2010 Memorandum	2010
Health Professions Regulatory Bodies Bill, 2010 Memorandum	2010
Bills	
Health Institutions and Facilities Bill, 2010	July 2010
Health Professions Regulatory Bodies Bill, 2010	July 2010
Report and Memorandum	
Report of the Parliament Select Committee on Health	October 2012
Council of Elders of the Pharmaceutical Society of Ghana memorandum to the Parliamentary Select Committee on Health on the Health Institutions and Facilities Bill, 2010	1 March 2011
Review of the Pharmacy Council Component (Part Four) of the Health Professions Regulatory Bodies Bill 2012 by the Pharmaceutical Society of Ghana (PSGH)	2012
Acts	
Health Institutions and Facilities Act 829	2011
Health Professions Regulatory Bodies Act 857	2013

The Acts were passed by the Parliament of Ghana and therefore the parliamentary debates reports (Hansards), which are verbatim records, were a great source of information on policy actors involved and how they framed and debated issues. Hansards transcripts are important data sources and provide a way to investigate how decisions are made [18]. Other documents generated outside the Parliament such as the Parliament Select Committee on Health report and memoranda submitted to the committee were also reviewed and analysed to map up discussions and decisions outside of the Parliament. The Health Institutions and Facilities Bill and Health Professions Regulatory Bodies Bill and corresponding Act 829 and Act 857 were also reviewed to trace content changes relating to pharmacy premises and business.

Key informant interviews were conducted to further understand the decision-making processes, policy actors’ role and framing of issues as well as triangulate data from the document review and analysis. The interviews were conducted in person and by phone by AK and RSB in Accra. Key informants were selected on the basis of their availability, experiences and knowledge of the decision-making processes, framing of issues and the policy actors involved and were informed of the purpose of the study and introduced to the research team. Key informants provided written and verbal consent before the commencement of the interviews. Eight respondents were interviewed in English between 28 November 2018 and 3 May 2019 and these included the executive secretary of the Pharmaceutical Society of Ghana (PSGH), former chief pharmacist of the Ministry of Health and past president of the PSGH (2011–2015), two former Pharmacy Council chairpersons (1994–2009; 2009–2015), former chief medical officer of the Ministry of Health (2002–2008), head of monitoring and evaluation of the Ministry of Health (2004 to date), former registrar of the Pharmacy Council (1981–1997) and the founding dean of the School of Pharmacy (University of Ghana). Five interviews were audio recorded and later transcribed. Notes were taken for three interviews when permission to audio record was not granted. The notes taken were verified by the respondents. The interviews on average lasted 50 min and the main questions were: Which actors have been involved in the decision-making and design processes of Acts 829 and 857? How did they frame issues and what narratives did they push forward to influence decisions? How was the content of the Acts designed and agreed upon? Key informants were informed of the documents reviewed and additional relevant documents sought from them to substantiate their information. Interviews were stopped when respondents provided no new information on pharmacy premises and business regulation decision-making processes, actors involved and how they framed issues. Personal identifiers of key informants were removed from the results session and referred to as KI 1, KI 2, etc. to ensure anonymity.

### Data analysis

Drawing on the power approaches to decision-making [10], we listed and categorized specific actors (whether as individuals or groups) with interest in pharmacy premises and business licensing on the basis of their power sources, roles, narratives, bureaucratic and political arrangement, and professional associations. Decision-making venues (within Parliament and the health sector bureaucracy) and processes including problem definitions and framing and timelines relating to discussions on the Health Professions Regulatory Bodies and Health Institutions and Facilities Bills in and out of Parliament were mapped out. The content on pharmacy premises and business licensing was traced from the Health Professions Regulatory Bodies and Health Institutions and Facilities Bills and Acts 829 and 857. The Bills submitted to Parliament and the amendments made over time were manually highlighted, mapped and compared to Act 829 and Act 857, respectively, and inclusions and deletions noted. Accompanying explanations and justifications for the deletion from the Bills and inclusions into the Acts from the Hansard transcripts, reports and memoranda were manually traced and documented.

Interview transcripts were read, analysed and manually organized into retrievable sections based on the research questions. Analyses of parliamentary debates, reports, memoranda, Bills and Acts were used to further corroborate information from our key informants. The transcriptions and document review and analysis notes generated were further coded using the following themes: policy actors and power source, framing narratives, Act 829 and Act 857.

Data were finally mapped out to chronological present timelines, decision-making processes (including problem definitions) and venues, actors with their power sources involved and the way they framed issues for pharmacy premises and business regulation.

## Results

### The Health Institutions and Facilities Act 2011, (Act 829)

The Minister for Health (with political power) submitted the Health Institutions and Facilities Bill (dated 23 July 2010) to Parliament on 28 October 2010 for first reading of the Bill [19]. The Ministry of Health and its agencies and stakeholders such as the Attorney’s General Office (principal legal advisers) drafted the Bill.

‘*The Health Institution and Facilities Bill was developed by the Ministry of Health and its agencies such as the Private Hospitals and Maternity Homes Board.*’ (KI 6: 29/11/2018)

Part 1 of the Bill sought to provide for a Health Facilities Regulatory Agency to license facilities for the provision of public and private health care services. The Health Facilities Regulatory Agency was to replace and expand the mandate of the Private Hospitals and Maternity Homes Board under Act, 1958 (No. 9) [20]. The Private Hospitals and Maternity Homes Board (Act 1958) was outdated and did not adequately regulate all health care facilities.

‘*The Private Hospital and Maternity Homes Board was over stretched and could not adequately regulate all private hospitals and maternity homes. The Act has not been revised since [it was] developed [in]1958 and a lot has happened since then within the health sector.*’ (KI 4: 20/01/2019).

‘*The Private Hospital and Maternity Homes Board’s objectives were outdated, and its regulation excluded other facilities such as eye care clinics, geriatric homes and diagnostic imaging technology clinic many of which were springing out in the country.*’ (KI 6: 29/11/2018)

The Bill therefore sought to fill in the gap created by the Private Hospitals and Maternity Homes Board (1958) and mandate the Health Facilities Regulatory Agency to license the operation of these practices: ‘*medical and dental services, clinics and hospitals, optometry and optician services, chiropody, convalescent and nursing homes, community health services, geriatric homes, nursing care, nursing agencies, maternity homes, occupational therapy services, physiotherapy services, dental laboratory technology services, clinical and bio-medical laboratory technology services, ophthalmic nursing services and physician assistants clinics*’ [20]. After first reading of the Health Institutions and Facilities Bill, 2010 in Parliament, the speaker of Parliament in accordance with Article 106 of the Constitution of Ghana referred the Bill to the Parliament select Committee on Health for consideration [19].

#### Parliament Select Committee on Health deliberations and framing outside Parliament

According to the 22 March 2011 parliamentary debates report, the Committee on Health requested for written memoranda on the Bill from the general public and stakeholders to engage them in the decision-making process. The Committee met for 3 days with those who presented memoranda and other stakeholders in the health sector to examine the Bill in detail [21]. The Committee reviewed the Bill and considered pharmacies as facilities to be licensed by the Health Facilities Regulatory Agency.

‘*During the Committee’s consultative meetings there were discussions of adding pharmacies to list of facilities to be regulated by the Health Facilities Regulatory Agency.*’ (KI 4: 21/01/2019)

‘*The Pharmacy Council had successfully regulated pharmacies over the decades and agenda of the Bill was to replace the Private Hospital and Maternity Board policy and certainly not to take over the regulatory mandate of the Pharmacy Council*’. (KI 2: 14/12/2018)

The stakeholders that met with the Committee to discuss the Health Institution and Facilities Bill are summarized in Table 2 [21]. Of these stakeholders, a Pharmacy Interest Group of the PSGH—the Council of Elders—had concerns about the discussion to include pharmacies in the facilities to be licensed by the Health Facilities Regulatory Agency and therefore sent a memorandum dated 1 March 2011 to the Committee.

**Table 2:** Stakeholders that discussed the Health Institutions and Facilities Bill, 2010 with the Parliament Committee [21]

Minister for Health, Hon Joseph Yieleh Chireh
Deputy Minister for Health, Hon Robert Joseph Mettle-Nunoo
The Acting Chief Director Ministry of Health, Dr. Sylvester Anemana
Chief Executives, Registrars and Directors of Agencies and Departments of the Ministry of Health
Society of Private Medical and Dental Practitioners of Ghana
Ghana National Chemical Sellers Association
The Pharmacy Interest Group of the Pharmaceutical Society of Ghana (Council of Elders)
Officials from the Attorney General’s Department (The Legislative Drafter—Principal Legal Adviser)

In the memorandum, the Council of Elders made the following submissions in relation to the intended addition of pharmacies to the First Schedule of the Health Institutions and Facilities Bill. One, ‘*it is the practice in most part of the world for a separate and independent authority to regulate both pharmacy practice including practitioners and licensing of pharmacy premises. This and the fact that under the existing legislation the Pharmacy Council is performing its mandate well and may have informed the decision to exclude pharmacies from the list of premises indicated in the Bill’.* Two*, ‘the Minister of Health’s memorandum to the Health Institution and Facilities Bill as published in the Gazette did not mention pharmacy at all’.* Three, ‘*currently the Pharmacy Council regulates about 12,000 registered facilities in Ghana made up of pharmacy retailers, wholesalers, retailers/wholesalers and manufacturing wholesalers and 10,000 chemical sellers. In addition, Pharmacy Council inspectors pay working visits to public hospitals pharmacies and dispensaries.*’ Four, ‘*for effective and efficient inspection and monitoring of activities in these premises, the Pharmacy Council has set up offices throughout the country. Except for the northern part of Ghana (Northern, Upper East, Upper West) which has a zonal office at Tamale, the rest of the country has regional offices located in the capitals namely Accra, Kumasi, Sekondi, Cape Coast, Koforidua, Ho and Sunyani*’.

#### Framing narratives and decisions relating to pharmacy premises regulation in Parliament (legislative decision-making venue)

During the second reading of the Health Institutions and Facilities Bill, the chairman of the Parliament Select Committee on Health (with legislative power) in his report to Parliament on 22 March 2011 noted that the Bill will expand the scope and mandate of the Private Hospital and Maternity Homes Board to regulate public health facilities as well as pharmacies. On the floor of Parliament, the Committee among other issues recommended adding ‘*pharmacies and chemical shops*’ to the definition of practice under clause 24 of the Bill [22]. But a parliamentarian was against the inclusion and noted that ‘*the regulation and licensing of pharmacy practice is not bundled up with other healthcare practices in most parts of the world. For instance, the General Pharmaceutical Council in the United Kingdom and State Boards of Pharmacy and the General Pharmacy Council in Nigeria are all responsible for the license of pharmacies and related premises and regulation of pharmacy practitioners*’. The parliamentarian further reiterated that the Pharmacy Council should be allowed to continue regulating pharmacies. The Minister for Health who is a parliamentarian supported this call to exclude pharmacies and the need to follow best practices around the world and allow the Pharmacy Council to continue its work. The Minister for Health noted that ‘*pharmacies were left out by [the] promoter of the Bill for good reasons*’ and urged members to vote for the motion to exclude pharmacies. The speaker called for a vote on the motion. The question was put, and motion agreed to maintain a list that excludes pharmacies [22].

On 23 June 2011, the Bill was discussed in Parliament during the consideration stage. Discussions focused on operationalising the activities of the Health Facilities Regulatory Agency and fine-tuning its functions. A member of the Parliament Select Committee on Health reiterated that ‘*the Bill gives the Health Facilities Regulatory agency the mandate to determine locations of both public and private health facilities including where district hospitals should be located*’. Using similar logic, he argued that the agency should determine where pharmacies should be located since they are health facilities. The motion to amend the list of facilities to be regulated by the agency was made and the amendment agreed to [23].

‘*Out of Parliament, the pharmacy fraternity were taken by a storm with the deliberations in Parliament to include pharmacies. This dramatic turnaround of events meant the advocacy and lobbying of the PSGH was not taken into account. Pharmacies were added to the list on the floor of Parliament*’. (KI 2: 14/12/2019)

The Health Institutions and Facilities Bill was read the third time and passed on 20 July 2011 [24]. The approved Health Institutions and Facilities Bill was gazetted on 31 December 2011 as Act 829 [8]. The changes relating to pharmacy premises made to the Health Institutions and Facilities Act 829, 2011 are summarized in Table 3 [8, 20].

**Table 3:** Summary of provisions (text) modifications relating to pharmacy premises in Act 829 [8, 20]

Health Institutions and Facilities Bill, 2010	Modified in Act 829
Object and functions of the Agency: Clause 3 (1) ‘*The object of the Agency is to license facilities for the provision of public and private health care services*’	Object of the Agency: Clause 3 ‘*The object of the Agency is to license and monitor facilities for the provision of public and private health care services*’
Facilities to be licensed: Clause 10.(1) ‘A person shall not operate a private facility unless the facility is licensed under this Act’ 10.(2) ‘*A person shall not operate equipment for a service specified in the First Schedule unless the facility in which the person operates is licensed under this Act*’	Facilities to be licensed: Clause 11.(1) ‘*A person shall not operate a facility unless the facility is licensed under this Act*’ *11.(2) ‘A person shall not operate equipment in a facility specified in the First Schedule unless the facility in which the person operates is licensed under this Act*’
Interpretation: Clause 24 ‘*Practice includes medical and dental services, clinics and hospitals, optometry and optician services, chiropody, convalescent and nursing homes, community health services, geriatric homes, nursing care, nursing agencies, maternity homes, occupational therapy services, physiotherapy services, dental laboratory technology services, clinical and bio-medical laboratory technology services, ophthalmic nursing services and physician assistants clinics*’	Interpretation: Clause 25 ‘*Practice includes medical and dental services, clinics and hospitals, services in pharmacies and chemical shops, optometry and optician services, chiropody, convalescent and nursing homes, community health services, geriatric homes, nursing care, nursing agencies, maternity homes, occupational therapy services, physiotherapy services, dental laboratory technology services, clinical and bio-medical laboratory technology services, ophthalmic nursing services and physician assistants clinics*’
First schedule The following facilities shall be licensed under this Act (a) Medical and dental (clinics and hospital) (b) Eye care clinics (c) convalescent and nursing homes (d) Geriatric homes (e) Maternity homes (f) Occupational therapy clinics (g) Physiotherapy clinics (h) Dental technology laboratory (i) Clinical and bio-medical laboratory (j) Medical assistant clinics (k) Diagnostic-imaging technology clinics (l) Osteopathy clinics (m) Prosthetics and orthotics clinics (n) Any other health care clinic or premises that may be determined by the Minister	First schedule The following facilities shall be licensed under this Act (a) Medical and dental (clinics and hospital) (b) Eye care clinics (c) Convalescent and nursing homes (d) Geriatric homes (e) Maternity homes (f) Occupational therapy clinics (g) Physiotherapy clinics (h) Dental technology laboratory (i) Clinical and bio-medical laboratory (j) Medical assistant clinics (k) Diagnostic-imaging technology clinics (l) Pharmacies and chemical shops (m) Osteopathy clinics (n) Prosthetics and orthotics clinics (o) Any other health care clinic or premises that may be determined by the Minister

### Health Professions Regulatory Bodies Act, 2013 (Act 857)

The Ministry of Health drafted the Health Professions Regulatory Bodies Bill, 2010 in consultation with professional regulatory bodies and associations (technical and profession knowledge) and the Attorney General’s office (principal legal advisers) to consolidate existing laws because they are similar in nature.

‘*The Ministry of Health in 2010 started a process to consolidate all laws regulating health professions into one Act of Parliament*’. (KI 5: 20/02/2019)

Although the Health Professions Regulatory Bodies Bill was dated 21 July 2010, same as the Health Institutions and Facilities Bill, it was submitted to Parliament on 4 March 2011 for first reading 8 months later.

‘*The Bills were drafted around the same time but passing the Health Institutions and Facilities Bill into law was a top priority since public health facilities and some private facilities were not regulated and the law regulating private hospitals and maternity homes was outdated.*’ (KI 6: 29/11/2018)

The Health Professions Regulatory Bodies Bill, 2010 sought to establish Allied Health Professions Council, Medical and Dental Council, Nursing and Midwifery Council, Pharmacy Council and to provide for related purposes. Part four of the Bill expanded regulations under the existing Pharmacy Act 1994. The Bill establishes the Pharmacy Council and its functions included ‘*register practitioners and license premises in the public and private sectors*’ (under clause 69c) and ‘*monitor and inspect pharmacy premises and other premises where pharmaceutical care is provided*’ (under clause 69e) [25]. After the first reading in Parliament by the Minister for Health on 4 March 2011, the Bill was referred to the Parliament Select Committee on Health for consideration [26].

#### Parliament Select Committee on Health deliberations and framing outside Parliament

According to the Committee’s report dated October 2012 requests were made for written memoranda on the Bill. The Committee had several meeting with stakeholders to examine the Bill in detail and these stakeholders are listed in Table 4 [26].

‘*The Committee’s meetings with stakeholders were protracted because of the many conflicting issues raised by existing professional regulatory bodies and associations. Among others, were disagreements as to which practitioners constitute an allied health professional and the inclusion of a new entity, the Psychology Council*.’ (KI 7: 28/11/2018)

**Table 4:** Stakeholders that discussed the Health Professions Regulatory Bodies Bill with the Parliament Committee [26]

Deputy Minister for Health, Hon Robert Joseph Mettle-Nunoo
The former Minister for Health, Hon Joseph Yieleh Chireh
The Medical and Dental Council
The Pharmacy Council
The Nurses and Midwives Council
The Allied Health Task Force
The Ghana Health Service
The Psychologists Associations of Ghana
The Law and Development Associates
Officials of the Ministry of Health
Officials from the Attorney General’s Department (The Legislative Drafter—Principal Legal Adviser)

The Committee proposed amendments to the Health Professions Regulatory Bodies Bill, 2010 and the amendments relating to pharmacy premises are listed in Table 5 [26].

**Table 5:** Proposed amendments by the Parliament Committee in relation to pharmacy premises [26]

Clause No. (Health Professions Regulatory Bodies Bill)	Proposed amendment
Clause 69 (Functions of the Council)	Paragraph (e) line 1 delete ‘pharmacy premises and other practices’ and insert ‘pharmacy practices and’
Clause 83 (Licensing of premises)	Delete ‘(1) A person shall not supply restricted medicines from premises unless the premises are licensed in accordance with this Part (2) A person who seeks to license premises for pharmacy practices shall apply to the Registrar in a manner determined by the Board (3) The Board may revoke a licence if satisfied that the physical conditions of the premises have ceased to be suitable for the supply of restricted medicines (4) A person who supplies restricted medicines from licensed premises shall notify the Board of material alterations in the structure of the premises within six months of the alteration (5) The licence for premises may be general or limited and is valid for the period determined by the Board (6) A general licence shall be issued for the supply of all classes of medicines and a limited licence shall be issued for the supply of medicines other than prescription only medicines and pharmacy only medicines.’
Clause 97 (Entry of premises)	Paragraph (a) line 2 delete ‘the licence of premises’ and insert ‘pharmaceutical company’
Clause 99 (Power of closure)	Subclause (1) line 3 delete ‘or where the premises are unlicensed’

#### Framing narratives and decisions relating to pharmacy premises and business regulation in Parliament

On 24 October 2012, the Deputy Minister for Health moved for the Health Professions Regulatory Bodies Bill, 2010 to be read a second time in Parliament [27]. The chair of the Committee on Health supported the motion and presented the committee’s report to Parliament. Proposed amendments related to licensing of Pharmacy premises presented to Parliament were as follows: One, ‘*delete pharmacy premises from Section 69 and replace with pharmacy practice*’. Two, ‘*delete the whole Section 83—licensing of premises’.* Three, ‘*under entry of premise section delete the licence of premises and replace with pharmaceutical company*’. Four, ‘*under power of closure section delete where the premises are unlicensed*’ [27].

‘*Clearly the Committee on Health sought to remove any provisions relating to licensing of pharmacy premise from the Health Professions Regulatory Bodies Bill.*’ (KI 4: 21/01/2019)

The Health Professions Regulatory Bodies Bill, 2011 was put forward for consideration on 25 October 2012 [28] and 18 December 2012 [29] and on both days no reference was made to pharmacy premises and business regulation as other parts for the Bill were discussed. However, on 20 December 2012, during the consideration stage, the chair of the Committee on Health stated that the Pharmacy Council no longer regulates pharmacy premises because of the Health Institutions and Facilities Act passed in July 2011. Therefore, the Health Professions Regulatory Bodies Bill, 2011 must be amended to avoid a conflict of who is the legitimate regulator. To this a member of Parliament responded and noted that amending the Bill was important for future interpretation of the Act should any issue arise in the courts [30].

The chair of the Committee on Health moved for clause 77 titled ‘supervision of pharmacy’ be deleted. Clause 77 states that ‘*A person shall not open or permit any other person to open premises to the public under the description of ‘pharmacy’, ‘dispensary’, ‘chemist’, ‘drug store’ or any other similar description unless a registered pharmacist is on the premises to supervise the dispensing of the medicines or medication’.* He noted that clause 77 would create confusion and contradiction since the Pharmacy Council will not regulate premises. This motion was contested by a parliamentarian who argued that clause 77 does not entrust the Pharmacy Council with power to license but the clause is a prohibitive provision to make room for creating an offence. In a rebuttal, the immediate past Minister for Health and a parliamentarian informed the House that the whole clause has been moved to clause 100 (titled offences) where it becomes a subclause. A motion was therefore passed for the amended order to stand as part of the Bill [30].

The following changes were also agreed to by parliamentarians. One, the deletion of clause 83 (licensing of premises); two, amendment to clause 84 (licensing of corporate bodies) and three, amendment to clause 97 (entry of premises). However, a request to delete the text ‘*where the premises are unlicensed*’ from clause 99 (power of closure) was contested and not agreed by Parliament. Clause 99 [1] states that ‘*An inspector may close premises that sell or supply restricted medicines where there are grounds to believe that a health hazard may exist on the premises or where the premises are unlicensed*’. The immediate Minister for Health opposed and stated the Pharmacy Council can inspect the licence of pharmacy premises issued by a different authority and that the Council will not demand facilities obtain the licence from them [30].

**Fig. 1:**
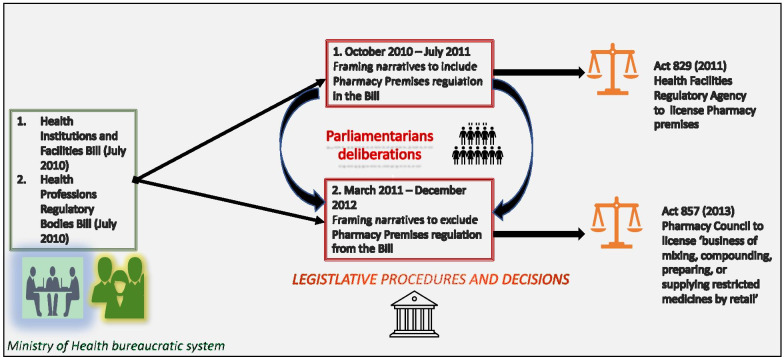
Summary of decision-making processes and venues for Act 829 and Act 857

The Health Professions Regulatory Bodies Bill 2011 went through two consideration stages on 21 December 2012 and pharmacy premises and business were not discussed [31, 32]. The Bill was read a third time and passed on 21 December 2012 [32]. The Pharmacy Act 1994 (Act 489) [7] was repealed and replaced by part four of the Health Professions regulatory bodies Act, 2013 (Act 857) [1]. Table 6 [1, 25] summarizes the main contents modified and maintained in the Health Professions Regulatory Bill, 2010 in relation to pharmacy premises and the business of mixing, compounding, preparing or supplying restricted medicines by retail. Figure 1 illustrates the decision-making processes and venues for Act 829 and Act 857.

**Table 6:** Summary of provisions (text) relating to pharmacy premises and business of retail in Act 857 (2013) [9, 25]

Health Professions Regulatory Bodies Bill, 2010 provisions modified or deleted in Act 857	Provisions of the Health Professions Regulatory Bodies Bill, 2010 maintained in Act 857	Provisions excluded in Bill, 2010 but included in Act 857
Functions of the Council: Clause 69 (c) ‘*register practitioners and licence premises in the public and private sectors*’ modified to Functions of the Council: Clause 80 (c) ‘*register practitioners*’	Functions of the Council: Clause 69 (Bill) and Clause 80 (Act 857) ‘*(d) ensure the equitable and accessible distribution of pharmaceutical premises*’	*License for wholesale supply of restricted medicines:* Clause 95 (1) ‘*A person shall not carry on the business of the wholesale supply of restricted medicines unless that person has a licence for the wholesale supply of restricted medicines.*’ (2) ‘*The Board may grant a licence for the wholesale supply of restricted medicines subject to conditions which may prohibit or limit the supply of restricted medicines of a particular description.*’ (3) ‘*A promotional or marketing office where a person intends to engage in the wholesale pharmacy business shall be licensed and supervised by a registered pharmacist*’
Functions of the Council: Clause 69 (e*) ‘monitor and inspect pharmacy premises and other premises where pharmaceutical care is provided*’ modified to Functions of the Council: Clause 80 (e) ‘*monitor and inspect pharmacy practice where pharmaceutical care is provided*’	Licensing of corporate bodies: Clause 84 (Bill) and Clause 93 (Act 857) ‘(1) *The Board may grant a licence to a body corporate or a government institution if satisfied that (a) the applicant is fit to carry on the business of mixing, compounding, preparing or supplying restricted medicines by retail, and (b) the business of the applicant is carried on under the supervision of a superintendent pharmacist*’	
Licensing of corporate bodies: Clause 84 ‘(1b) *the applicant’s business is carried on under the supervision of a superintendent pharmacist*’ *modified to* Licensing of corporate bodies: Clause 93 ‘*(1b) the business of the applicant is carried on under the supervision of a superintendent pharmacist*’	Power of closure: Clause 99 (Bill) and Clause 108 (Act 857) ‘(1) *An inspector may close premises that sell or supply restricted medicines where there are grounds to believe that a health hazard may exist on the premises or where there are unlicensed*’	
Entry of premises: Clause 97 ‘*(a) to inspect the registration of a pharmacist, pharmaceutical care providers or the licence of premises*’ modified to Entry of premises: Clause 106 ‘*(a) to inspect the registration of a pharmacist, pharmaceutical support staff or pharmaceutical company*’	Regulations: Clause 101 (Bill) and Clause 111 (Act 857) ‘*(e) prescribe conditions including the type of premises for the issue of general and limited licence of the Council*’	
Supervision of pharmacy: Clause 77 *‘A person shall not open or permit any other person to open premises to the public under the description of ‘pharmacy’, ‘dispensary’, ‘chemist’, ‘drug store’ or any other similar description unless a registered pharmacist is on the premises to supervise the dispensing of medicines or medication’* moved to Offence: Clause 110 (c) and text maintained	Interpretation: Clause 102 (Bill) and Clause 112 (Act 857) *‘‘premises’ includes pharmacy premises or other facility authorized for practitioners under this Part and a pharmacy department of a hospital, clinic, a house, building, structure, tent, caravan, land, ship, boat, an aircraft mechanically propelled device and other place or facility in which pharmaceutical services are offered*.’	
*Licensing of premises:* Clause 83—Deleted		

## Discussion

The study highlights the varied roles policy actors played in shaping the content of Act 829 and Act 857 in different decision-making venues and processes and the politics of regulation. The Ministry of Health and its technical actors drafted the Health Institutions and Facilities and Health Professions Regulatory Bodies Bills within its bureaucratic system with input from its agencies such as the Pharmacy Council and the Private and Maternity Homes Board. In the Ministry of Health, the Bills were agreed upon by the formulators. Content contestations started when the Bills were discussed in boarder stakeholder engagements and venues with actors with conflicting goals and vested interest. First, the Bills were read in Parliament at separate periods and referred to the Committee on Health for further interrogation and deliberations. The committee members interrogated the Bills and invited the Ministry of Health and other stakeholders for discussions to better understand the Bills and take decisions on the provisions proposed. Although the Pharmacy Council and PSGH made their suggestions relating to pharmacy premises regulation, the ultimate decision rested with the parliamentarians. The parliamentarians took the final decisions on the content of the Bills and other stakeholders such as PSGH and the Pharmacy Council and could not intervene in decisions relating to pharmacy premises regulation on the floor of Parliament.

Decision-making context, processes and venue determine which actors are allowed to participate and made decisions [33]. The contexts of policy choice influence options and actions and are thus important in understanding emergence and unfolding of laws making [33]. Though the Bills’ formulators and internal stakeholders such as the Pharmacy Council and PSGH agreed on the initial provisions of the Health Institutions and Facilities and Health Professions Regulatory Bodies Bills, the dramatic turn of events occurred in a venue that they had no control over and could not participate and make their submissions. Decisions and suggestions made outside of Parliament may be relevant and technically sound; however, the ways in which parliamentarians framed and pushed pharmacy premises regulation issues during proceedings were important and influenced decisions. Parliamentarians as powerful actors were able to interpret issues from their understanding and convinced others [11]. The Parliament Select Committee on Health as proponent of inclusion of pharmacy premises on the list of facilities regulated by the Health Facilities Regulatory actively participated in debates and moved for motions to support their recommendations based on their reasoning and understanding of issues relating to pharmacy premises. As noted in another study [34], law-makers sense making of a bill (to be enacted into an act) was influenced by their own understanding of details of specific sections and categories of the Bill and their ability to influence others [34]. Therefore beyond the legislative power of law-makers, law-makers use their narratives and understanding to lend legitimacy and attract support [35].

Legislative powers are vested with parliamentarians and exercised in accordance with the Constitution of Ghana [36]. Parliamentarians debated the principles [36] of the Health Institutions and Facilities Bill and the Health Professions Regulatory Bodies Bill through the legislative processes [36]. As part of the legislative procedure, the Committee involved other policy actors in the decision-making process. Although the PSGH through the Council of Elders made their suggestions relating to pharmacy premises regulation, the ultimate decision rested with the parliamentarians. The parliamentarians took the final decisions on the basis of consensus and non-legislative actors such as PSGH could not intervene in the framing narratives and decisions relating to pharmacy premises regulation and business on the floor of Parliament. This finding of decision-making venue restricting participation and influence is similar to the restrictive nature of the business meetings conducted as part of the Ghanaian health sector institutionalised policy-making processes [37]. Health sector policy decisions are restricted to key elites—policy actors with prerogative authority to formulate policies and those with financial resources [37].

The Committee on Health constantly framed pharmacy premises as a health care facility and promoted this labelling and the need for regulation by the Health Facilities Regulatory Agency. This framed narrative was supported in Parliament and common narratives can be supported for different reasons and varied interest [11]. However, pharmacy premises are indeed health facilities and there were no contentions with this fact. Since Health Institutions and Facilities Bill and Health Professions Regulatory Bodies Bill were developed concurrently, the formulators did not foresee the challenge and the unintended effort that labelling of pharmacies as health facilities could present. Part four of the Health Professions Regulatory Bodies Bill expanded the existing Pharmacy Act 1994 (Act 489) and these amended expansions did not consider potential conflict with the Health Institutions and Facilities Bill. Conflict in pharmacy regulation may be attributed to a process of repeated amending of existing regulations without due consideration to the impact that such amendments have [38]. This, however, was not the case here; the Bills were in tandem and complementary and not a mere repetition of existing laws. In Parliament, the Bills were discussed separately, and this synergy was not realised. Timing of discourse and decisions made in the design of these Acts therefore played an important role in how the issue of pharmacy premises was later framed and discussed.

### Policy and practice implications

The way in which law-makers framed and designed the policies to regulate pharmacy premises and the business of mixing, compounding, preparing or supplying restricted medicines by retail has implications for policy and practice. One, is the potential duplication of services and efforts by these regulatory institutions. Two, is the creation of a monitoring hurdle. Persons in the business of supplying restricted medicines by retail do operate pharmacies and may repudiate a licence altogether and play chase with both agencies. Three, pharmacies and persons in the business of supplying restricted medicines by retail can obtain licences from the Health Facilities Regulatory Agency and the Pharmacy Council, respectively, and operate in the same vicinity and compete for clients. Four, the reversal of consensus reached on the Bills could cause tension between the Parliament Select Committee on Health on one side and the Pharmacy Council and the PSGH on the other. Finally, the ceding of Pharmacy Council’s mandate of licensing pharmacy premises to a new agency could create some ambiguity within the pharmacy fraternity. Pharmacy practice could therefore be faced with uncertainty as individuals would have to decide whom to seek a licence from to operate pharmacy premises and supply restricted medicines by retail.

This potential overlap can be mitigated by Parliament amendment to Act 829 and Act 857. The Pharmacy Council can be (re)mandated to license pharmacy premises as pharmacies are in the business of supplying restricted medicines by retail. Alternatively, the granting of a licence to a body corporate or a government institution to carry on the business of mixing, compounding, preparing or supplying restricted medicines by retail under the supervision of a superintendent pharmacist can be ceded to the Health Facility Regulatory Agency. In either way, there are no straight answers and the decision lies with parliamentarians. The potential challenge of the passage of these laws such as overlaps of regulatory roles, fragmentation of regulation, extra cost to practitioners and pharmacies among others could offer a window of opportunity [39] for policy revision. The implementations of Acts 829 and 857 are not evaluated and therefore research is needed to investigate their impact on pharmacy practice in Ghana. Findings from this implementation study can serve as evidence to support revisions of the Acts or otherwise and present a window of opportunity for re-engagement of major policy actors to take a second look at the unfolding development in the greater public interest or maintain the status quo.

### Study strengths and limitations

The study relied on varied data sources and methods to corroborate findings. Parliament proceedings which provided information on how decisions relating to pharmacy premises and business regulations were made in Parliament were an important data source; however, these Hansards do not capture who said what and to whom during backbench discussions and these can be opportunities to influence others and create alliances. During the research period, efforts were made to interview parliamentarians involved in direct discussions either on the floor of Parliament or as members of the Parliament Select Committee on Health to further understand their individual framing of issues, but this was unsuccessful. We acknowledge this difficulty and therefore triangulated data from multiple sources in an effort to present the decision-making processes and policy actors involved in the formulation of Act 829 and Act 857.

## Conclusion

The final content of the Health Institutions and Facilities Act and Health Professions Regulatory Bodies rested with the parliamentarians (with legislative power). Decision-making venues and processes limited participation and input from other policy actors, although these non-legislative actors had some level of influence. While framing and labelling of policy issues are important tools in decision-making, the timing and venues of framing and labelling are equally vital. Powerful legislative actors ultimately determined which statutory regulatory body regulates pharmacy premises and the business of mixing, compounding, preparing or supplying restricted medicines by retail. Implementation of these policies could create overlap with implications for policy and practice. Additional research is needed to assess the impact of these Acts on pharmacy practice in Ghana and this can serve as bargaining evidence for reforms or otherwise. As legislative processes and the health sector bureaucratic system may be similar in other LMICs, we hope this paper contributes to learning and the formulation of pharmacy premises and business regulation laws.

## Data Availability

Data sharing is not applicable to this article as no datasets were generated or analysed during the current study.

## Abbreviations

HeFRA: Health Facilities Regulatory Agency
PSGH: Pharmaceutical Society of Ghana
LMICs: Low- and middle-income countries
